# The Relationship between Poisson’s Ratio Index and Deformation Behavior of Asphalt Mixtures Tested through an Optical Fiber Bragg Grating Strain Sensor

**DOI:** 10.3390/ma15051882

**Published:** 2022-03-03

**Authors:** Xu Liu, Mo Zhang, Wanqiu Liu

**Affiliations:** 1 Fundamental Research Innovation Center, Research Institute of Highway, Ministry of Transport, 8 Xitucheng Road, Beijing 100088, China; sikui2003@outlook.com; 2 Department of Civil Engineering, Tsinghua University, 30 Shuangqing Road, Beijing 100084, China; 3 School of Civil and Transportation Engineering, Hebei University of Technology, 5340 Xiping Road, Tianjin 300401, China; 4 College of Civil Engineering and Architecture, Hainan University, 58 Renmin Avenue, Haikou 570228, China; liuwanqiu@dlut.edu.cn

**Keywords:** asphalt mixture, flow-rutting, axial and lateral deformation, optical fiber Bragg grating

## Abstract

Flow-rutting is the main distress leading asphalt pavement to undergo premature maintenance, and is produced by the rapid accumulation of shear deformation in asphalt layers under high temperature and heavy loads. The excessive permanent deformation of the asphalt mixture at high temperature is related to the decrease of the material’s stability during the temperature increase and an unfavorable stress state, e.g., low confining pressure and high shear stress, which eventually leads to significant nonlinear viscoplastic behavior. In this research, dynamic modulus tests and repeated loading tests were carried out at 35 °C and 50 °C to analyze the deformation response of materials under a strain amplitude of <200 με and 400~500 μεs, respectively. Based on the in-lab repeated loading tests, the total deformation of the asphalt mixture in each loading and rest cycle was divided into three parts, being elastic, viscoelastic, and viscoplastic strain, and the measurement of the axial and lateral strain of cylindrical samples was realized with the aid of optical fiber Bragg grating strain sensors. It was found that the experimental index of the ratio between lateral strain and longitudinal strain (RLSLS), derived, but distinguished, from Poisson’s ratio defined limited in elastic strain, can characterize the deformation in viscoelastic and viscoplastic behaviors of the mixes. Furthermore, the indices of dynamic modulus, phase angle, complex Poisson’s ratio, stiffness, and creep rate of four types of mixes containing different volcanic ash fillers and asphalt binders at 35 °C and 50 °C were systematically analyzed by the jointed experiments of modified dynamic modulus tests and repeated loading tests, and their consistent trending to the RLSLS index was obtained.

## 1. Introduction

Flow-rutting (FR) is one of the most harmful forms of distress on high-grade asphalt pavement. It is a type of unrecoverable shear deformation resulting from the creeping flow of the asphalt binder and the rearrangement of aggregate particles in the mesoscale of the mix [1,2], which accumulates rapidly in the asphalt layer under repeated vehicle loads. FR is highly dependent on the properties of the material and greatly impacted by environmental temperatures and traffic loading. In particular, the FR disease becomes more significant under conditions of high temperature and heavy wheel loads, which can result in destructive impacts on the service lives of pavements. Due to serious rutting induced by overload states, many asphalt pavements in China have to be greatly repaired or rebuilt within two to three years, far from the designed service life of approximately 15 to 20 years [3]. From the perspective of pavement design, this phenomenon is due mainly to the mismatch between the evaluation method of material performance and the formation mechanism of FR.

Based on the relationship between the material and structure of asphalt pavement, the permanent shear deformation in asphalt layers is usually introduced by the coupling effects of the following factors: (1) high-temperature conditions that induce more significant viscoplastic behavior of the asphalt mixture and results in higher permanent deformation; (2) heavy wheel loads that can lead to larger shear stress that then intensify the viscoplastic flow; and (3) the stiffness constitution in the structural layers of pavement can result in an adverse combination of principal stresses (e.g., low average stress or large deviator stress) and decrease the plastic yielding threshold of hot mixed asphalt (HMA).

In respect to material performance improvement, many scholars have tried to introduce new materials into the mixture design to comprehensively improve the high temperature performance of asphalt mixtures [4,5,6,7,8,9,10,11]. However, precise evaluation methods and indicative indices are still needed to reflect the development of permanent deformation within asphalt pavement under high temperature and complex loading conditions to further improve and predict the performance and stability of an asphalt mixture. Empirical and theoretical assessments are the two major classes of evaluation methods of rutting. The Marshall test and wheel tracking test are the most widely applied empirical laboratory testing methods to reflect the high-temperature failure of HMA at a specific temperature and loading mode [12,13,14,15]. However, they cannot completely simulate the working states of real pavement, such as continuously varied loads and temperatures. Therefore, field accelerated loading tests, such as accelerated loading facility and accelerated pavement testing, are used to investigate permanent deformation and establish a life-cycle prediction model of rutting [16,17,18]. However, theses field accelerated loading tests would be hugely expensive to perform for large-scale road tests and the testing can only be carried out for certain structural compositions of pavement.

Furthermore, there is always a theoretical gap between empirical evaluation indices and HMA performances, leading to difficulties in performing an accurate analysis. Mechanical models and testing indices are important for evaluating deformation properties of asphalt binders and mixtures. To better understand the states of permanent deformation, Lagos-Varas et al. developed a viscoelastic model using derivatives of fractional order, which can describe the creep, recovery, and relaxation behaviors in an asphalt mixture [19,20]. With the increasing application of viscoelastic and viscoplastic theories in the mechanical analysis of asphalt mixtures, temperature-controlled dynamic triaxial tests are becoming important evaluation methods [21], and the related repeated load permanent deformation (RLPD) test is the most recommended evaluation method [22,23]. The periodical loads that consist of a half-sine loading period and a rest period in each cycle can well represent the complex loading condition, and the calculated flow number (FN) and the deductive FN index can effectively evaluate the permanent deformation of the HMA [22,23,24,25]. The mixture samples have to be continuously loaded until the accelerated shear failure stage to obtain the FN value, which needs a long loading period that is sometimes not achievable. Rather than a slow and constant development, the corresponding FR permanent deformation is a fast increase of shear deflection, which is generated by nonlinear mechanical behaviors, mainly viscoplastic and plastic deformation, which are hard to quantitatively evaluate by the FN or FN index.

In this study, the ratio of lateral strain to longitudinal strain (RLSLS) tested in the RLPD test was adopted to elucidate the significance of viscoplastic and plastic behavior of HMA in the FR. RLSLS was proposed based on the modification of Poisson’s Ratio (PR), which is a traditional concept in elastic theory. As a more generic index, RLSLS can better reflect the complex elastic-visco-plastic behavior of HMA, while the accurate measurement of lateral and longitudinal strain is necessary. However, the common deformation sensor, linear variable differential transformer (LVDT) that adhered to HMA samples, is prone to soften under high temperatures, which affects the testing accuracy. To overcome this problem, an optical fiber Bragg grating (OFBG) strain sensor was adopted to measure the lateral and longitudinal strain of cylindrical HMA specimens in this study. The standard dynamic modulus (DM) tests, modified DM tests and RLPD tests were applied at 35^°^C and 50^°^C, determined by consideration of test procedures and sensor range, in order to analyze the change characteristics of the mechanical parameters of the asphalt mixture when the ambient temperature was transformed from a medium temperature to a higher temperature. First, four types of HMA with two types of VA fillers and two types of asphalt binder, i.e., base asphalt and SBS-modified asphalt, were investigated in this study. The HMA samples were tested by applying a haversine load in an uniaxial direction at the loading frequencies of 1, 0.5, and 0.1 Hz to (i) explore the feasibility of the OFBG strain sensor in the measurement of complex Poisson’s ratio (CPR) in modified DM tests; and (ii) analyze the trend of CPR to evaluate the high-temperature stability of HMA. Second, based on RLPD testing results, different types of HMA were compared with respect to the stiffness and rate of permanent strain (RPS). In addition, the total strain of HMA in a single loading-unloading-rest period was divided into three parts: instantaneous recoverable strain, delayed recoverable strain, and residual strain. By calculating the RLSLS corresponding to these strains, the effects of different types of VA and asphalt binders on deformation behavior were analyzed and the correlation between RLSLS and the shear permanent deformation of the HMA was discussed.

## 2. Methodology

### 2.1. Materials

The research group of this study used porous volcanic ash (VA) to replace traditional mineral powder as a fine filler in the asphalt mixture. It was found that the mastics and HMA with the combination of VA-and SBS-modified asphalt had better performance on both high- and low-temperature properties [10,11]. The representative types of HMA and their compositions are detailed as follows:

#### 2.1.1. Volcanic Ash Fine Filler and Asphalt Binder

**Figure 1:**
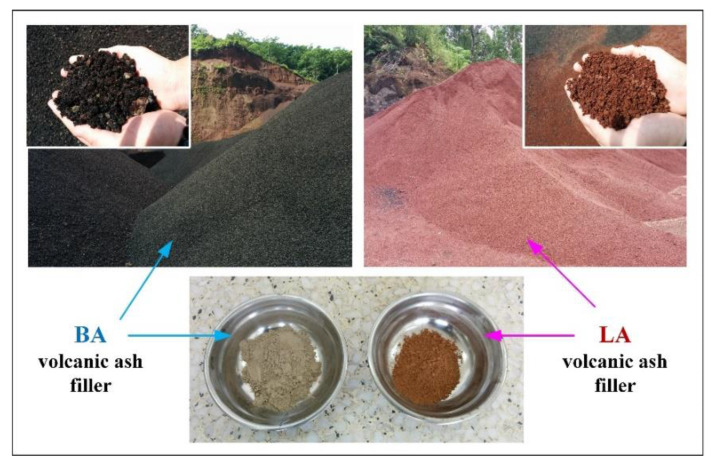
The volcanic ash fine filler of Bahaozha (BA) and Lazihe (LA).

The two types of VA were collected from the Lazihe and Bahaozha areas in the Jilin province of China, and are denoted by LA and BA, respectively (as shown in Figure 1). They were ground to particle sizes smaller than 0.075 mm and used as fine fillers in the HMA, of which the physical properties are listed in Table 1. LA and BA have similar apparent densities, while LA had a larger average particle size and higher specific surface area than BA. According to a previous study [11], the LA particles have a richer porous structure than BA. In addition, BA has a smoother particle surface than LA, based on scanning electron microscopy (SEM) characterization.

On the other hand, base asphalt and 5 wt% of SBS-modified asphalt with a penetration level of 90 (0.1 mm) were adopted as two types of asphalt binders, which were denoted by P and S, respectively. The properties of the base asphalt were measured following the ASTM Standards, as shown in Table 2.

#### 2.1.2. Asphalt Mixture

In this study, four types of HMA with different combinations of the two types of asphalt binder (base/SBS) and two types of volcanic ash fine fillers (LA/BA) were investigated, as listed in Table 3. The aggregate gradation in Table 4 was used for all of the HMA samples to eliminate the impacts of aggregates. An asphalt/aggregate mass ratio of 4.6%, determined by the Marshall method [26], and a fine filler to asphalt binder ratio of 1:1 were used for all the sample sets. The samples were designated by “type of VA-type asphalt binder”, which were LA-S, LA-P, BA-S, and BA-P, respectively. Cylindrical HMA samples, with a diameter of 100 mm and height of 150 mm, were prepared with a gyratory compactor for the standard DM tests, modified DM tests, and the RLPD tests in this study. All the specimens were cured in an environmental chamber at a specific temperature for 4 h before testing.

### 2.2. Optical Fiber Bragg Grating Strain Sensor

The optical fiber Bragg grating (OFBG) sensing technique has been used in experimental research on pavement material and structure, which can bear a high compaction force and high temperatures during the testing process [27,28]. In the modified DM tests and RLPD tests, an OFBG strain sensor was adopted to measure the longitudinal and lateral strain of HMA cylindrical samples, with the highest sampling frequency of 300 Hz and largest strain capacity of 10,000 με. Due to its high ductility and highest accuracy of 1 με, OFBG was allowed to attach to the surrounding cylinders with the closest contact to measure the lateral strain of the HMA under dynamic loading.

**Figure 2:**
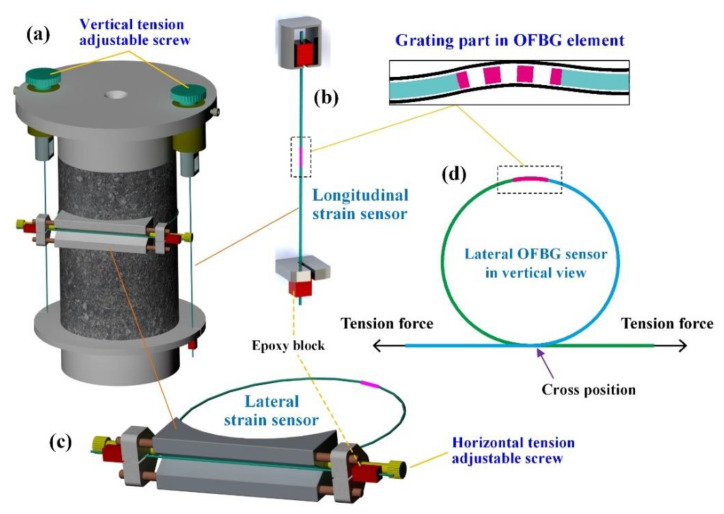
Schematic setup of (**a**) the cylindrical HMA sample in DM tests with two (**b**) vertical OFBG sensors and one (**c**) horizontal OFBG sensor, in which the (**d**) OFBG element was fixed with two epoxy resin blocks and stretched with two screwing nuts.

With particularly fabricated fastening equipment, three sets of OFBG sensors were aligned surrounding the cylindrical samples, of which two were aligned in the vertical direction and one was aligned in the horizontal direction, as schematically shown in Figure 2. At the two sides of the grating section in each OFBG sensor, two epoxy resin blocks were fixed and connected to the adjusting screws, with which the OFBG was stretched during testing to ensure consistent deformation between the sensor and samples. The OFBG was stretched to a strain value slightly higher than 50 με in the horizontal direction and approximately 5000 με in the vertical direction. The average reading of the two vertical sensors was used to calculate the longitudinal strain, while that of the horizontal sensor was the circumferential strain used to obtain the lateral strain.

The linear measurement of the strain by the OFBG was realized based on the reflecting wavelength change in the grating section of optical fiber [27], of which the strain can be calculated by Equation (1):

(1) $$\Delta \varepsilon =\frac{1}{1-P_{e}}\left(\frac{\Delta \lambda_{1}}{\lambda_{1}}-\frac{\Delta \lambda_{2}}{\lambda_{2}}\right)$$

where Δε is the strain of the OFBG, $P_{e}$ is the stress optical coefficient, $\lambda_{1}$ is the Bragg wavelength of grating areas when stress and temperature both changed, and $\lambda_{2}$ is the Bragg wavelength of grating areas when only temperature is changed.

The longitudinal and lateral strain of the cylindrical sample were converted from the sensor reading according to the geometric correlation, as shown in Equations (2) and (3):

(2) $$\varepsilon_{long}=\frac{\varepsilon_{olong}\cdot l_{o}}{h}$$

(3) $$\varepsilon_{lat}=\frac{\varepsilon_{olat}\cdot l_{o}}{\pi d}$$

where $\varepsilon_{long}$ and $\varepsilon_{lat}$ are the longitudinal and lateral strain of the sample, respectively, $\varepsilon_{olong}$ and $\varepsilon_{olat}$ are the longitudinal and lateral strain of the OFBG, respectively, in mm/mm, $l_{o}$ is the length of the OFBG between the two epoxy resin blocks, in mm, and h and d are the height and diameter of the sample, respectively, in mm.

### 2.3. Dynamic Modulus Tests

#### 2.3.1. Standard Dynamic Modulus Test

The dynamic modulus (DM) and phase angle (PA) of the HMA at temperatures of 35 °C and 50 °C, and frequencies of 25 Hz, 20 Hz, 10 Hz, 5 Hz, 1 Hz, 0.5 Hz, and 0.1 Hz were tested with a standard dynamic modulus test to evaluate the medium- to high-temperature performance of the materials. The 35 °C and 50 °C conditions were two representative temperatures selected from the middle-to high-temperature section in the temperature range of −10 °C~54 °C, specified in AASHTO TP62-2007 [29], used to analyze the change characteristics of material parameters in the process of temperature increase. For each type of asphalt mixture, four groups of parallel specimens were prepared for testing, and the mean value of the results was taken as the representative value of the material. The loading was conducted using a universal testing machine (UTM) with a maximum load capacity of 100 kN. During testing, the contact pressure of 5 N was retained between the UTM loading cell and the top surface of the cylindrical sample. The testing temperature was pre-adjusted to, and retained at, 35 °C and 50 °C with the embedded environmental chamber.

#### 2.3.2. Modified Dynamic Modulus Test

The modified DM test is a test method for the exploration of applying the OFBG as a deformation sensor based on the standard DM test. The traditional LVDT strain measuring apparatus in the standard DM test was replaced by an OFBG strain sensor in the modified DM test to simultaneously measure the axial and lateral strain of the cylindrical samples, while the testing temperature, materials, and loading equipment remained the same. The material properties at higher temperatures can be characterized at low testing frequencies according to the time-temperature superposition principle (TTSP) [30]. Therefore, the relatively low testing frequencies of 1 Hz, 0.5 Hz, and 0.1 Hz were adopted for the performance analysis of the mixtures, at which permanent deformation was more prone to develop. In this part, two sets of parallel specimens were tested for each type of HMA.

**Figure 3:**
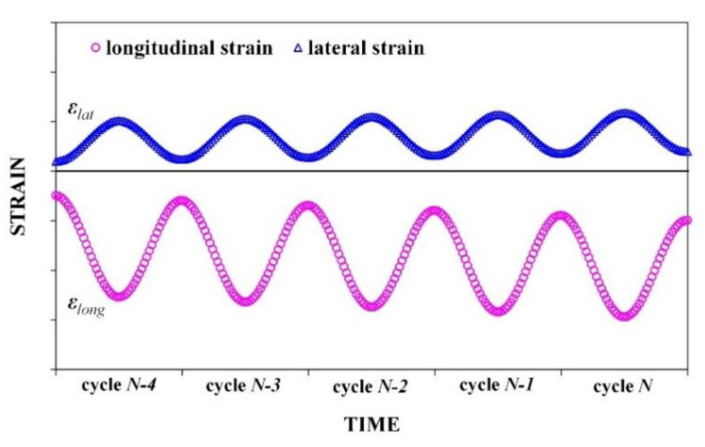
Longitudinal and lateral strain in the last five cycles obtained by OFBG sensors.

The amplitude of the strain wave of the HMA specimen was limited in the linear viscoelastic range (<200 με) during testing. The longitudinal strain was negative since the specimen was compressed, while the lateral strain was positive. As shown in Figure 3, the strain–time curves in the last 5 loading cycles were obtained and fit using Equations (4) and (5). The curve fitting process and parameter calculation were accomplished with MATLAB code.

(4) $$\varepsilon_{long}\left(t\right)=\left(-1\right)\times \left[\varepsilon_{0,long}\sin\left(2\pi ft+\varphi_{long}\right)+a_{c,long}\left(t\right)+Y_{long}\right]$$

(5) $$\varepsilon_{lat}\left(t\right)=\varepsilon_{0,lat}\sin\left(2\pi ft+\varphi_{lat}\right)+a_{c,lat}\left(t\right)+Y_{lat}$$

where f is the loading frequency, t is the testing time, $\varepsilon_{0}$ and φ are the amplitude and phase angle of the strain curve, respectively, while $\varepsilon_{0}$ is positive, Y is the vertical compensation during curve fitting, $a_{c}\left(t\right)$ is the creeping rate, and the subscript long and lat represent the longitudinal and lateral directions, respectively.

In addition, the complex Poisson’s ratio (CPR) and related mechanical parameters were calculated by using Equations (6) to (8):

(6) $$\nu^{*}=\frac{\varepsilon_{lat}\left(t\right)}{\varepsilon_{long}\left(t\right)}=\left|\nu^{*}\right|\cdot e^{-i\delta_{\nu }}$$

(7) $$\left|\nu^{*}\right|=\frac{\varepsilon_{0,lat}}{\varepsilon_{0,long}}$$

(8) $$\delta_{\nu }=\varphi_{long}-\varphi_{lat}$$

where $v^{*}$ is the CPR, $\left|v^{*}\right|$ is the normal of $v^{*}$, which was named the dynamic Poisson’s ratio (DPR), $\delta_{\nu }$ is the phase lag of lateral strain compared to longitudinal strain, and $0\leq \delta_{\nu }\leq \frac{\pi }{2}$.

### 2.4. Repeated Load Permanent Deformation (RLPD) Tests

#### 2.4.1. Testing Procedure

The RLPD tests were conducted to investigate the development of axial and lateral deformations of the HMA specimens under complex loading conditions by using different loading/rest time combinations at the two temperatures of 35 °C and 50 °C in order to compare with the results of the DM and modified DM tests. Limited by the strain range of the OFBG sensor, the 60 °C condition was not adopted as the experimental temperature, which was usually used in the traditional high-temperature performance evaluation test of asphalt mixtures. At 60 °C, the permanent deformation of HMA develops more rapidly than at lower temperatures, and the test data of enough load cycles cannot be obtained. Therefore, 50 °C is selected as the higher experimental temperature in this experiment, and can be comprehensively analyzed with the results of the DM tests and modified DM tests. Although 35 °C and 50 °C are not the extreme temperatures for high-temperature failure of HMA, the decay of material properties among these two temperatures can still reflect the high-temperature performance of the materials.

An unconfined axial periodical load was applied on the HMA specimens in RLPD tests to further emphasize the viscoelastic and viscoplastic behavior of asphalt mixtures. Each loading cycle consisted of one section of haversine wave and one resting section, i.e., loading/rest process. In each loading period, the load amplitude of 500 N and 400 N were applied at 35 °C and 50 °C, respectively, to retain the strain amplitude in the range of 400~600με. The strain level exceeds the linear viscoelastic domain of the HMA [31,32] to further intensify the permanent deformation of materials compared to the modified DM tests. Due to the strain limit of the OFBG sensor, 100 and 50 loading cycles were selected for the temperature conditions of 35 °C and 50 °C, respectively. Three groups of loading/rest time combinations, ① 0.1 s/0.9 s, ② 1 s/1 s, and ③ 1 s/9 s, were adopted in this experiment, and the pairwise comparison was used to reflect the influence of loading time, rest time, and loading frequency on the deformation features of the material. The loading mode of 0.1 s/0.9 s was used for the specification AASHTO TP79 [33], and another two comparison groups were proposed on this basis. For different materials, two sets of parallel experiments have been carried out.

#### 2.4.2. Data Analysis

The stiffness and permanent deformation rate of different HMA samples under repeated loading/unloading conditions were analyzed. Their differences in high-temperature stability and permanent deformation resistance were compared to investigate the synergistic effect of volcanic ash and the SBS modifier.

**Figure 4:**
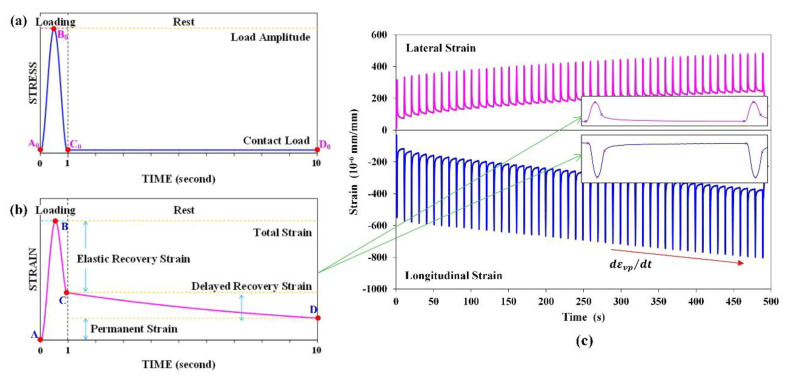
(**a**) Loading curve and (**b**) corresponding strain curve in the loading/rest combination of 1 s/9 s in RLPD tests, and (**c**) an illustration of the longitudinal and lateral permanent strain rate.

In each loading/rest time cycle, as shown in Figure 4, the loading curve and corresponding strain curve in the loading/rest time combination of 1 s/9 s were plotted as an example. As the curve of one periodic loading cycle plotted in Figure 4a, Point A_0_ was the initial point of one loading period, B_0_ was the peak point of the haversine loading, C_0_ was the ending point of the haversine loading (i.e., the initial point of the rest time), and D_0_ was the ending point of one periodic loading cycle. The loading curve A_0_B_0_C_0_D_0_ can be divided into three phases: the loading phase of A_0_B_0_, the unloading phase of B_0_C_0_, and the rest time phase of C_0_D_0_. As shown in Figure 4b, the corresponding strain curve ABCD was also divided into four sections: (1) total strain $\varepsilon_{total}$ (phase AB); (2) instantly recoverable strain $\varepsilon_{e}$ (phase BC); (3) delayed recoverable strain $\varepsilon_{ve}$ (phase CD); and (4) residual strain $\varepsilon_{vp}$. Point A corresponded to the starting point of one loading cycle; Point B was the peak strain point, the time that was very close to that of the peak point B_0_ on the haversine loading curve; Point C was the ending point of the applied haversine curve; and Point D was the ending point of this loading cycle. The four strain portions have the relationship shown in Equation (9).

(9) $$\varepsilon_{total}=\varepsilon_{e}+\varepsilon_{ve}+\varepsilon_{vp}$$

where $\varepsilon_{total}$ is the total strain, $\varepsilon_{e}$ is the elastic strain, $\varepsilon_{ve}$ is the viscoelastic strain, and $\varepsilon_{vp}$ is the residual strain. It should be noted that the viscoplastic strain and plastic strain were not separated in the analysis and are represented by $\varepsilon_{vp}$.

The total strain was divided into three parts of instantaneous recoverable strain, delayed recoverable strain, and residual strain to characterize the elastic, viscoelastic, and viscoplastic behaviors, respectively, of the HMA with different VA fillers and asphalt binders. The axial and lateral strain in 50 cycles of the loading/rest (1 s/9 s) process at 50 °C is shown in Figure 4c. With MATLAB code, for each loading/rest cycle, the starting point *A_n_*, strain magnitude point *B_n_*, starting point of rest *C_n_*, and ending point of rest period *D_n_*, which was also the starting point of next loading/rest cycle *A_n_*_+1_, were obtained to analyze the different strains with Equations (10)–(14). The rate of permanent strain (RPS) was used to characterize the resistance of permanent deformation of the materials since the developing trend of the strain curve in each loading/rest cycle was determined by residual strain.

(10) $$\varepsilon_{total,n}=\varepsilon_{B,n}-\varepsilon_{A,n}$$

(11) $$\varepsilon_{e,n}=\varepsilon_{B,n}-\varepsilon_{C,n}$$

(12) $$\varepsilon_{ve,n}=\varepsilon_{C,n}-\varepsilon_{A,n+1}$$

(13) $$\varepsilon_{vp,n}=\varepsilon_{A,n+1}-\varepsilon_{A,n}$$

(14) $${\frac{d\varepsilon_{vp}}{dt}|}_{n}\approx \frac{\varepsilon_{A,n+1}-\varepsilon_{A,n-1}}{t_{A,n+1}-t_{A,n-1}}$$

where t is the time period, n is the analyzed loading cycle, N is the total loading cycle, which is 50 in the example shown in Figure 4c, $\varepsilon_{total,n}$ is the total strain in the *n*th cycle, $\varepsilon_{e,n}$ is the elastic strain (or instantly recoverable) part of $\varepsilon_{total,n}$, $\varepsilon_{ve,n}$ and $\varepsilon_{vp,n}$ are the viscoelastic strain (or delayed recovered strain) and viscoplastic strain (or residual strain) part, respectively, and ${\frac{d\varepsilon_{vp}}{dt}|}_{n}$ is the mean RPS in these *n* cycles.

The average value of each index in the last 10 cycles was calculated for analysis. In addition, the stiffness and RLSLS of each type of HMA can be further calculated with Equations (15) and (16), respectively.

(15) $$S=\frac{\sigma_{amp}}{\varepsilon_{total}}$$

(16) $$v_{x}=\frac{\varepsilon_{x,lat}}{\varepsilon_{x,long}}$$

where $\sigma_{amp}$ is the amplitude of longitudinal stress, and the subscript x can be total, e, ve, or vp.

It should be noted that RLSLS is different from PR. PR is a concept in elastic theory, requiring the strain of materials to be within a linear elastic range. In this study, the strain magnitude of the HMA specimens in each loading cycle was approximately 500 με, which allowed materials to have more significant nonlinear behavior and a larger increasing rate of permanent strain.

In this study, the parameters of DM, PA, CPR, stiffness, and RPS for asphalt mixtures under the temperature conditions of 35 °C and 50 °C can be obtained by the DM test, modified DM test, and RLPD test, respectively, as shown in Table 5. Through the comprehensive analysis of multiple test parameters, the rationality of the test results can be verified with each other. Moreover, the performance of the materials can be evaluated from different perspectives. According to the experimental results, the change characteristics of the HMAs in the process of temperature increase were analyzed, and the performance difference of the material can be comprehensively evaluated. In addition, based on the strain analysis in the RLPD test, the elasto-visco-plastic behaviors of the mixtures during the deforming process can be further characterized to explore the relationship between the RLSLS index and the permanent deformation of the materials.

## 3. Results and Discussion

### 3.1. Standard DM Testing Results

**Figure 5:**
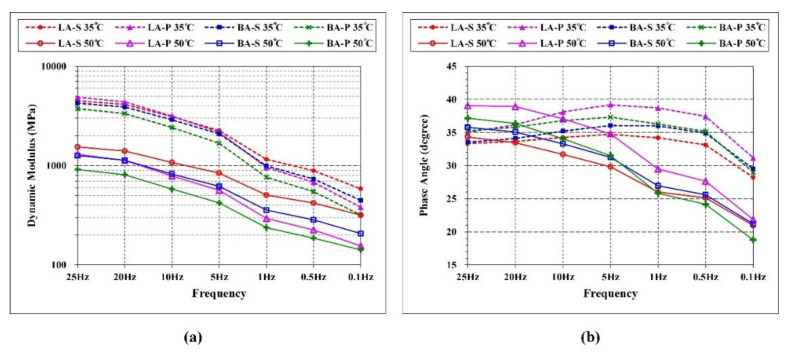
The dynamic modulus of HMA at 35 °C and 50 °C (**a**), and phase angle at 35 °C and 50 °C (**b**).

The dynamic modulus (DM) and phase angle (PA) of the four types of HMA were measured in standard DM tests [29] at 35 °C and 50 °C under seven different testing frequencies ranging from 0.1 to 25 Hz. The value range and frequency curve of the four materials are arranged in Table 6 and Figure 5 respectively. As shown in Figure 5a, the DM values of the four mixes tested at both 35 °C and 50 °C all decreased with a smaller testing frequency, which was equivalent to the increase in temperature according to TTSP [30]. The moduli of each mixture at 35 °C were greater than that at 50 °C. This decrease of DM at high temperatures was induced by the more significant behavior of viscosity flow within the asphalt binder or mastic, which led to a softening of the HMA. Therefore, a high DM indicated a more stable mesostructure composition of the mix. As a result, unrecoverable relocation or restructuration was less prone to occur in the aggregate skeleton of HMA.

On the other hand, the PA of all the mixtures at 35 °C increased from 25 Hz to 5 Hz and then decreased. In contrast, the PA of all the materials monotonically decreased with decreasing frequency at 50 °C, as shown in Figure 5b. This indicated that the temperature of approximately 35 °C was a transmission domain of the viscoelastic properties for these four types of materials. Therefore, the mechanical stability of the HMAs can be reflected by the 35 °C results. It should be noted that the tangent of PA represented the ratio between viscous loss energy and elastic storage energy. Low PA indicated more apparent elastic behavior, while high PA indicated a higher proportion of viscous behavior, which suggested more creep deformation occurrence. Furthermore, a smaller variation in PA under different frequencies at 50 °C also represented high stability, which can result in a higher rutting resistance.

The difference in testing results for the four types of HMAs were mainly induced by the different performances of asphalt mastic, which consists of VA filler and asphalt binder since the same aggregate gradation was applied. Based on the analysis of DM and PA, the performance of the four types of HMA at 35 °C can be ordered as LA-S > BA-S > BA-P > LA-P. This result indicated that the VA-SBS-modified HMA possessed better performance than the base asphalt mixture. Given that the temperature of 35 °C was not extremely high, the PA change at this temperature was more profound than DM change, which was more indicative of material stability. Since LA-P presented low DM and high change of PA, its performance was incomparable to the others. The performance of the four types of HMA at 50 °C presented a similar order to that at 35 °C, while LA-S had the most extraordinary high-temperature performance, followed by BA-S. Based on the DM and PA testing results, the two SBS-modified HMA presented better high-temperature performance than the other two that used base asphalt.

### 3.2. Modified DM Test

**Figure 6:**
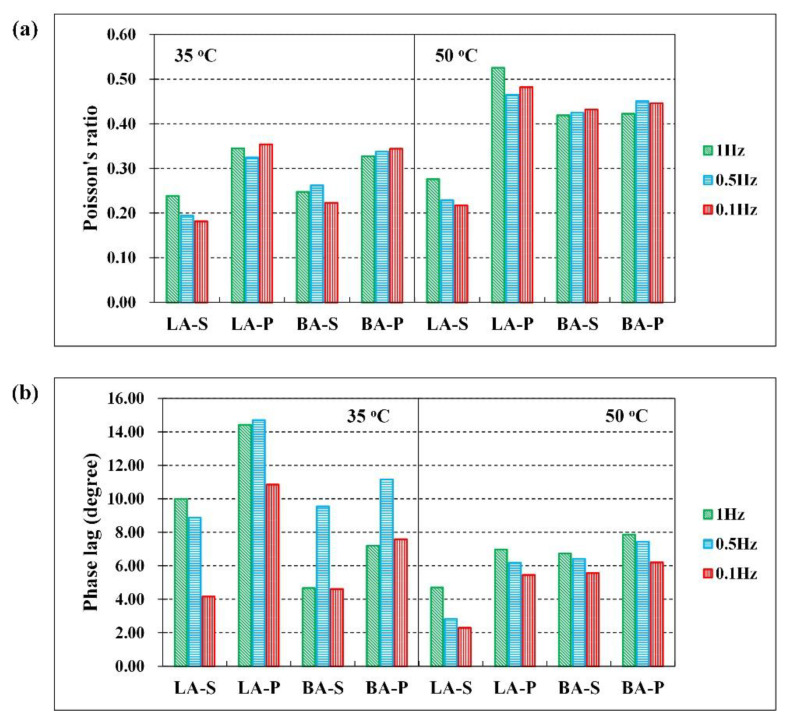
The DPR at 35 °C and 50 °C (**a**), and the phase lag of the mixes at 35 °C and 50 °C (**b**).

In the modified DM tests, the applicability of OFBG was explored in the measurement of the CPR of HMA cylindrical samples under harmonic loading. With the aid of OFBG sensors, the DPR ($\left|v^{*}\right|$) and the phase difference of the strain wave in the lateral and longitudinal directions of HMA, in terms of phase lag ($\delta_{\nu }$), were obtained at two temperatures (35 °C and 50 °C) and three frequencies (1 Hz, 0.5 Hz, and 1 Hz), as summarized in Figure 6 and Table 7. Similar to the analyses of DM and PA in the standard DM test, the testing results at 35 °C also can be used to assess the mechanical stability of the mixes, while those at 50 °C were applied in the investigation of the permanent deformation properties of HMA at high temperatures.

Based on the DPR and phase lag of the four HMAs shown in Figure 6a,b, the $\left|v^{*}\right|$ values of the two VA-SBS HMAs at 35 °C were in the range of 0.2~0.25, while that of the VA-base HMAs were approximately 0.35. According to the results at the three testing frequencies, the four types of mixtures were in the order of LA-S < BA-S < BA-P ≈ LA-P with respect to DPR. On the other hand, the correlation among the phase lags was more complex. LA-P was more distinguished, with $\delta_{\nu }$ being approximately 13 degrees, while the $\delta_{\nu }$ of the other three materials was approximately 8 degrees. Considering the tests at low frequencies that had more adverse impacts on permanent deformation, the phase lags of the HMA followed the ascending order of LA-S < BA-S < BA-P < LA-P. Combined with the DM and PA results in Figure 5, the temperature stability of HMA at 35 °C was in the opposite order of LA-S > BA-S > BA-P > LA-P, which illustrated that CPR contains $\left|v^{*}\right|$ and $\delta_{\nu }$ can be used to reflect the performance difference of the asphalt mixtures. The lower $\left|v^{*}\right|$ and $\delta_{\nu }$ of the HMA were, the better temperature stability and mechanical properties.

The $\left|v^{*}\right|$ and $\delta_{\nu }$ at 50 °C showed different trends compared to those at 35 °C, as shown in Figure 6. LA-S presented the most significant difference compared with the other HMAs in both DPR and phase lags. First, the $\left|v^{*}\right|$ of LA-S at 50 °C was slightly higher than that at 35 °C and still remained in the range of 0.2~0.3, whereas the $\left|v^{*}\right|$ of the other three mixes apparently increased from 35 °C to 50 °C, which were all larger than 0.4. The smaller change in DPR indicated that LA-S has more stable mechanical properties with the varying temperatures. Furthermore, a lower $\left|v^{*}\right|$ suggested a less incompressible viscous flow deformation and more recoverable elastic deformation of LA-S during the loading process. Second, LA-S had the lowest $\delta_{\nu }$ at 50 °C, which was approximately 4 degrees, while the $\delta_{\nu }$ of the other materials were similar to each other, approximately 6~8 degrees. Since a larger $\delta_{\nu }$ would lead to extra squeezing and abrasing process due to incoordination between the internal lateral strain and longitudinal strain, more serious permanent deformation would result. The above analyses suggested that the extraordinary high-temperature performance was presented by LA-S, while BA-S was only slightly better than the two types of base asphalt mixtures. The high-temperature performance still had the order of LA-S < BA-S < BA-P < LA-P based on DPR values.

Based on any of the four test indices of DM, PA, DPR, and phase lag, the performance of LA-S was the best. Furthermore, the variation amplitude of its indices were also the smallest during the temperature increase. Further, the other three mixtures also approximately satisfy this rule. This indicates that the high temperature performance of the HMA was intrinsically related to the stability of mechanical properties under temperature changes. In addition, the CRP or DPR index, which was related to the lateral deformation, indicated that the smaller the value, the better performance of the material. This indicated that a parameter of Poisson’s ratio also had the potential to be used as an evaluation index of material performance.

However, the strain level of HMA in the standard DM and modified DM tests was controlled under 200 με, which would not show significant permanent deformation. Therefore, the RLPD tests were conducted in this study to further analyze the mechanical properties of the HMA and their relevance to the RLSLS index, along with the development of permanent deformation.

### 3.3. RLPD Test

#### 3.3.1. Stiffness

**Figure 7:**
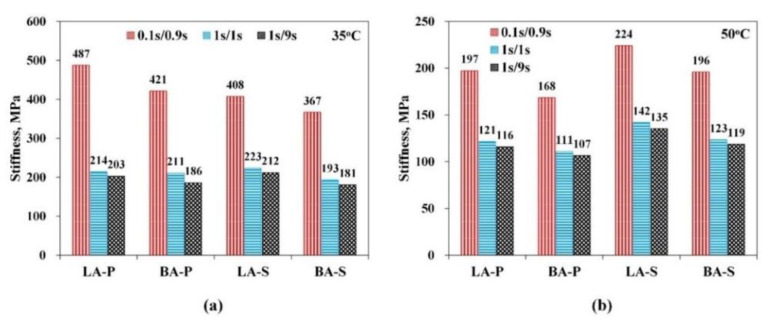
Stiffness of the four types of HMAs at (**a**) 35 °C and (**b**) 50 °C under the conditions of different loading/rest time combinations.

The stiffness of the HMA at specific temperatures, loads, and loading/rest time combinations was calculated using Equation (15) and compared to indicate the characteristics of mechanical responses of mixtures with different VA-binder mastics for multiple impact factors. The stiffness of the HMA samples at 35 °C and 50 °C are plotted in Figure 7.

First, the stiffness of all the mixtures at 35 °C under the condition of a short loading time per cycle (0.1 s/cycle) was approximately twice that of the stiffness under the other two loading conditions of 1 s loading time, while the stiffness under the long rest time per cycle (9 s/cycle) was slightly lower than that under the short rest time per cycle (1 s/cycle). The loading period had a significant influence on the experimental results. In addition, for the mixtures with the same asphalt binder, the LA filler HMA showed a slightly larger stiffness than the corresponding mix with BA.

Under the 50 °C condition, the effects of the three loading/rest time combinations on stiffness were the same as those at 35 °C, which also presented the order of $S_{0.1/0.9}>S_{1/1}>S_{1/9}$. However, when the temperature rose to 50 °C, the stiffness of the VA-base asphalt mixture was lower than that of the corresponding VA-SBS mixture, and the four materials showed an order of LA-S > BA-S ≈ LA-P > BA-P.

Since the same gradation was used for all the investigated mixes, it indicated that the viscoelastic properties of the inner mastic can influence the modulus level of the mixture at high temperatures. However, the trend shown by the stiffness of the HMA was not as significant as those of DM, PA, and CPR in the DM and modified DM tests. As a result, it was less applicable to demonstrate more subtle differences of material performance by the only index of stiffness.

#### 3.3.2. Rate of Permanent Strain (RPS)

**Figure 8:**
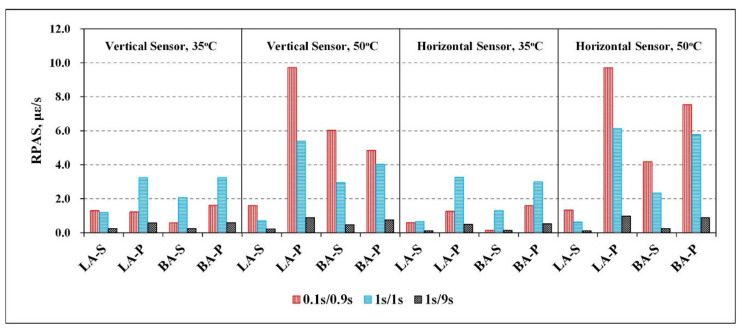
Longitudinal and lateral RPS of mixes at 35 °C and 50 °C under different loading/rest time combinations.

The longitudinal and lateral RPS of the four types of HMA at 35 °C and 50 °C were calculated with their vertical and horizontal strain data using Equation (14), as summarized in Table 8 and Figure 8. According to its definition of $\frac{d\varepsilon_{vp}}{dt}$, RPS can directly indicate the rate of development of permanent deformation at specific temperatures, loading magnitude, and loading/rest period.

As illustrated in Figure 8, the lateral and longitudinal RPS of the same HMA at 35 °C developed similar trends, which were significantly affected by loading/rest time combinations. The RPS in the combination of 1 s/1 s was apparently higher than the counterparts in the other two combinations, while the RPS in the 0.1 s/0.9 s combination was larger than that in the 1 s/9 s one, indicating that (i) permanent deformation was highly dependent on the maximum loading period; and (ii) a relatively long rest period can decrease permanent deformation of the HMA with a longer time of strain recovery. In general, the two types of VA-SBS mixtures have lower RPS than the other two VA-base mixtures at 35 °C, which followed the order of LA-S ≈ BA-S < LA-P ≈ BA-P.

For the results at 50 °C, both the lateral and longitudinal RPS followed the ascending order of LA-S < BA-S < BA-P < LA-P, elucidating that LA-S had the best resistance of creep deformation in the high-temperature condition, as shown in Figure 8. For the same type of HMA tested at 50 °C, the RPS in the 1 s/9 s loading combination was still the lowest. However, the RPS in the 0.1 s/0.9 s combination became the largest at 50 °C, different from 35 °C, at which the largest RPS value occurred in the 1 s/1 s combination. This indicated that the viscoelastic property of the mixtures significantly changed at a high temperature of 50 °C, which was more apparent and raised the sensitivity to the loading frequency variation. In general, the RPS at 50 °C was apparently higher than that at 35 °C, suggesting the more significant creep deformation of HMA under high-temperature conditions.

In general, the high-temperature performance of the HMAs interpreted by stiffness and PRS was consistent with that analyzed based on the indices of DM, PA, and CPR at the same temperature. This consistency indicated that the three sides of HMA, high temperature, mechanical properties, and deformation behavior, were the different expressions of the constitutive relationship of the material. Therefore, these experimental results were essentially determined by the mesostructure constitutions of the material, which was the theoretical foundation of studies for establishing the related indices of permanent deformation using mechanical parameters of the HMA.

#### 3.3.3. Ratio of Lateral Strain to Longitudinal Strain (RLSLS)

**Figure 9:**
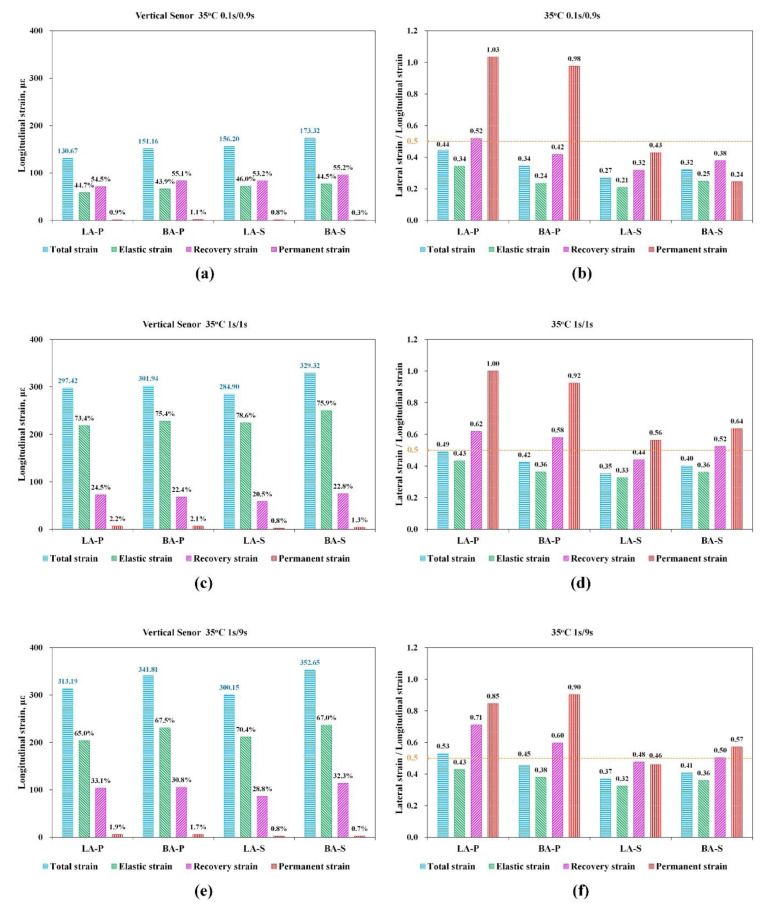
The longitudinal strain and the corresponding lateral/longitudinal strain ratios at the loading/rest time combinations of 0.1 s/0.9 s (**a**,**b**), 1 s/1 s (**c**,**d**), and 1 s/9 s (**e**,**f**) at 35 °C.

**Figure 10:**
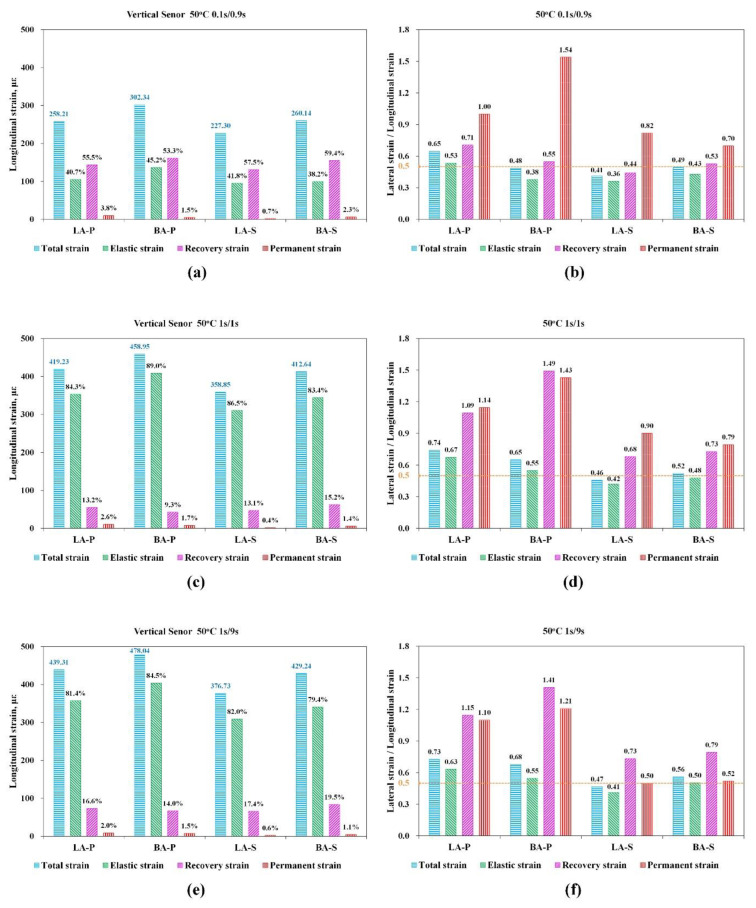
The longitudinal strain and the corresponding lateral/longitudinal strain ratios at the loading/rest time combinations of 0.1 s/0.9 s (**a**,**b**), 1 s/1 s (**c**,**d**), and 1 s/9 s (**e**,**f**) at 50 °C.

In the RLPD tests, the total strain of HMA in one loading/rest period was divided into instantaneous recovered strain, delayed recovered strain, and residual strain, according to Equation (9), to represent the elastic, viscoelastic, and viscoplastic behaviors, respectively. The longitudinal strains of all the HMA samples at 35 °C in different loading combinations are shown in Figure 9a,c,e, with their fractions in total strain marked. Correspondingly, the RLSLS values calculated with Equations (10)–(13), denoted by $v_{tatal}$, $v_{e}$, $v_{ve}$, and $v_{vp}$, were plotted in Figure 9b,d,f, respectively. The same types of results at 50 °C were plotted in Figure 10a–e.

##### Strains and RLSLS at 35 °C

As shown in Figure 9a,c,e, the total strains of HMA at the 0.1 s/0.9 s loading combination at 35 °C were all approximately 150με, which was nearly one-half of the strains under the 1 s/1 s and 1 s/9 s loading combinations. The fraction of elastic recoverable strain in total strain for the 1 s/1 s and 1 s/9 s loading combinations (approximately 65~75%) were apparently higher than the proportion of delayed recovered strain (approximately 20~35%). At a loading combination of 0.1 s/0.9 s, the fraction of elastic strain (~45%) was slightly lower than that of the viscoelastic strain (~55%). The proportion of viscoelastic strain was increased from 20% to 30% when the loading combination changed from 1 s/1 s to 1 s/9 s, indicating the delayed recovery property of asphalt mixtures. In general, the proportion of viscoplastic strain in the total strain of the three types of loading conditions were all low, in the range of 1~3%. The total strain of the four HMAs showed the relationship of LA-S < BA-S and LA-P < BA-P, while the permanent strain followed the order of LA-S ≈ BA-S < BA-P ≈ LA-P.

On the other hand, the differences of the HMA samples in the performance at 35^°^C can be more distinctly demonstrated with RLSLS. As shown in Figure 9b,d,f, most of the RLSLS values corresponding to the four types of strains were lower than 0.5. In all of the three loading combinations, the $v_{vp}$ of LA-P and BA-P exceeded 0.5 (approximately 1.0), and their $v_{ve}$ also had a larger value (approximately 0.5~0.7). The $v_{vp}$ and $v_{ve}$ of the other two HMAs of LA-S and BA-S were relatively low, in the range of 0.25~0.65. The LA-S had the lowest RLSLS value among the four materials, and its corresponding VA-SBS mix of LA-S was largest in the same loading combinations. In general, the VA-base asphalt mixtures presented a trend of $v_{e}<v_{ve}<v_{vp}$ and the VA-SBS mixtures had a trend of $v_{e}<v_{ve}\approx v_{vp}$. As a result, the differences of the four types of HMA at 35 °C all resulted in the change of RLSLS, which were lower while the corresponding HMA had better performance.

##### Strains and RLSLS at 50 °C

As illustrated in Figure 10a,c,e, the total strain of all of the HMAs at 50 °C were apparently higher than those at 35 °C, which was approximately 300 με under the 0.1 s/0.9 s loading condition, and exceeded 400 με when the loading conditions of 1 s/1 s and 1 s/9 s were applied. This result was closely related to the modulus decrease induced by the temperature increase. The elastic strain, $\varepsilon_{e}$, under the loading conditions of 1 s/1 s and 1 s/9 s both had higher proportions, larger than 80%, while that in the loading condition of 0.1 s/0.9 s was lower than 50%, which was attributed to the increase in the viscous response of the HMA in high loading frequencies. Comparing the testing results in loading conditions of 1 s/1 s and 1 s/9 s, the proportion of viscoelastic strain, $\varepsilon_{ve}$, for 1 s/9 s was slightly higher than that for 1 s/1 s. This resulted from the more delayed recovered strain of the HMA under a longer rest time. Similar to the 35 °C conditions, the viscoplastic strain, $\varepsilon_{vp}$, had a low proportion within the total strain, which was approximately 1~3%.

In addition, the RLSLS values of the four HMAs at 50 °C were also analyzed and were all higher than their counterparts at 35 °C. Noting that the $v_{ve}$ and $v_{vp}$ of mixtures at 50 °C were all larger than 0.5. The $v_{ve}$ and $v_{vp}$ of VA-base mixes were close to 1.5, significantly higher than those of VA-SBS mixes (0.5~0.9). This result indicated that the change in performance and mechanical properties of the HMA can be characterized with the RLSLS parameter. Moreover, the $v_{e}$ and $v_{ve}$ of the HMA under loading conditions of 1 s/1 s and 1 s/9 s were both slightly higher than those at 0.1 s/0.9 s. All of the asphalt mixtures showed a trend of $v_{e}<v_{ve}\approx v_{vp}$, similar to that at 35 °C. It can be concluded that the $v_{tatal}$, $v_{e}$, $v_{ve}$, and $v_{vp}$ of LA-S were the lowest, of which the high-temperature performance was also the best and the LA-P still obtained the worst result, which was the same as at 35 °C. As a result, the two types of VA-SBS HMA had lower RLSLS than the two types of VA-base mixtures.

As the analysis of RLSLS values of the four HMAs, when the more nonlinear behaviors introduced unrecoverable deformation of $\varepsilon_{ev}$ and $\varepsilon_{vp}$, the lower material parameters of $v_{x}$ would be obtained, which is consistent with the phenomenon shown by the CPR index in the modified DM tests.

##### Relationship between RLSLS and Permanent Strain

**Figure 11:**
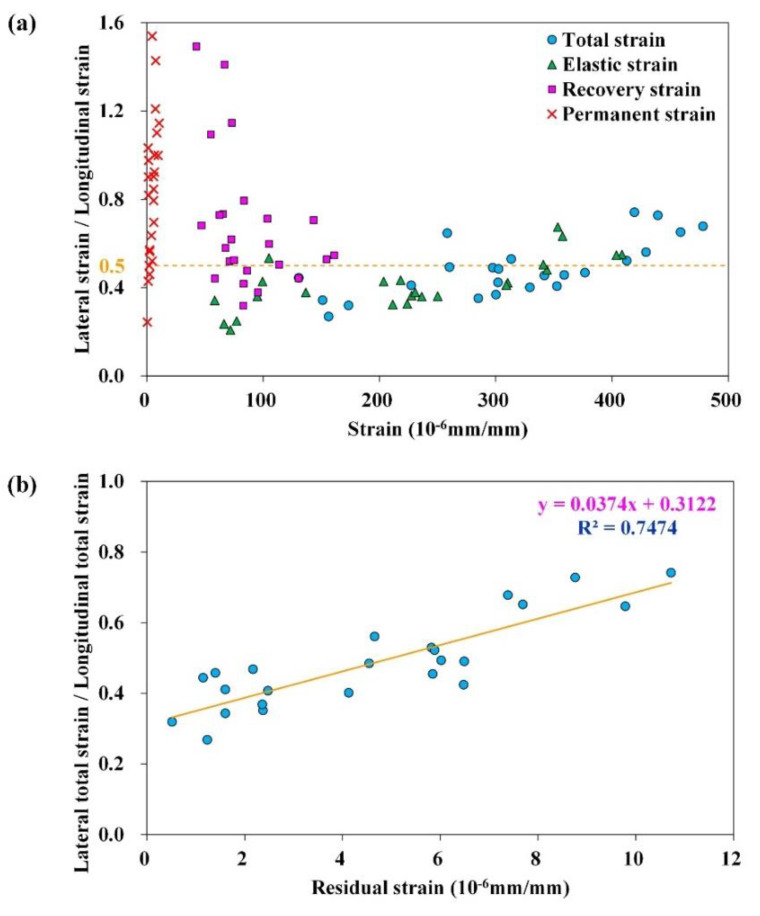
The (**a**) RLSLS value distribution of different strain types and (**b**) the linear fitting of $\nu_{total}$ and $\varepsilon_{vp}$.

According to the RLPD testing results at 35 °C and 50 °C, the RLSLS indices corresponding to $\varepsilon_{e}$, $\varepsilon_{ve}$, and $\varepsilon_{vp}$ were distributed in different value ranges, which followed the ascending order of $v_{e}$<$v_{ve}$<$v_{vp}$, as shown in Figure 11a. The $v_{e}$ was the lowest since the corresponding elastic strain was mainly characterized by the elastic behavior of the HMA, which was consistent with Poisson’s ratio of ideal elastic materials being smaller than 0.5. On the other hand, the $v_{vp}$, corresponding to the viscoplastic strain, represented unrecoverable viscoplastic and plastic defromation induced by the dislocation and reconstruction of the mastic and aggregate-skeleton, which were both uncompressible shear deformations. Although the theoretical upper limit of Poisson’s ratio was 0.5, the value of the RLSLS index was allowed to exceed 0.5 for the complex elastic-visco-plastic behavior of materials. These results were attributed to the viscous behavior induced by strain rate-relevant shear flow in asphalt binder or mastics, which was also a theoretically uncompressible shear deformation. Therefore, the numerical distribution of RLSLS for $v_{e}$, $v_{ve}$, and $v_{vp}$ corresponded to different mechanical behaviors and had apparent discrepancies during the deformation process of the HMA.

Therefore, this discrepancy can effectively characterize the permanent deforming property of the HMA. As illustrated in Figure 10b,d,f, the differences in $v_{ve}$ and $v_{vp}$ between VA-SBS mixtures and VA-base mixtures at 50 °C were more significant than the difference in $v_{e}$, indicating that (i) VA-SBS mixtures had better resistance to permanent deformation and a better deformation recovery property than VA-base mixtures; and (ii) this modification in mastic also decreased the proportion of viscous flow in the deformation process of HMA, presented by the lower $v_{ve}$ and $v_{vp}$ of LA-S and BA-S. Furthermore, LA-S had the lowest $v_{e}$, $v_{ve}$, $v_{vp}$, and $\nu_{total}$ of the four types of HMA, consistent with its better performance characterized by DM, PA, CPR, stiffness, and RPS parameters (as shown in Table 9) than the others. This indicated that the proportion of viscous and plastic behaviors of LA-S was relatively low, while the recoverable elastic and viscoelastic behavior was more significant during the deformation process.

Based on the above analyses, the $\nu_{total}$ obtained in the RLPD tests was the integral expression of elastic, viscoelastic, and viscoplastic behaviors of the HMA, which determined the development of permanent deformation of the materials. The residual strain and the corresponding RLSLS index, $\nu_{total}$, of the four HMAs at 35 °C and 50 °C are plotted in Figure 11b, of which two of the coefficient of determination (R^2^) values were approximately 0.75. This indicated that $\nu_{total}$ can elucidate the permanent deformation property of the HMA. It should be noted that this correlation was calculated based on the four HMAs containing both base asphalt and SBS-modified asphalt at 35 °C and 50 °C, which was generally applicable and not limited to particular types of binders and specific temperatures.

Therefore, the RLSLS index of $\nu_{total}$ obtained in the RLPD tests was a promising indicator for evaluating the flow-rutting investigated in this study. First, the external factors of temperature, loading, and confining pressure can be well controlled in RLPD tests, which significantly affects the development of permanent deformation. In this way, the performance of HMA can be evaluated based on the specific temperature and stress conditions of different pavement structures. Second, FR was generated by the fast accumulation of viscoplastic and plastic deformation in asphalt layers, which occurred in the complex conditions of adverse temperature and load. Therefore, the RLSLS index was highly accommodated to the generation mechanism of FR since it can characterize the mechanical behavior of the HMA during the deformation process. Finally, the total strain, corresponding to $\nu_{total}$, can directly indicate the performance of the HMA, rather than dividing total strain into three parts and calculating the corresponding RLSLS indices, which can further simplify the experimental assessment of asphalt mixtures.

In follow-up research, the rutting analysis of asphalt pavement with different thicknesses and material composition should be carried out based on the RLSLS index. The quantitative relationship between this index and the development of permanent deformation in a specific pavement structure under complex temperature field and stress field need to be explored, to establish a more effective evaluation method and prediction model for FR.

## 4. Conclusions

In this research, the axial and lateral deformation features of four HMAs, using two VA fillers combined with the binder of base asphalt and SBS-modified asphalt, were systematically analyzed at 35 °C and 50 °C through the DM test and RLPD test. The following conclusions can be drawn:

a. Based on the developed OFBG strain sensor, the high-frequency measurement of the axial and lateral strain of cylindrical HMA specimens under a dynamic loading mode was realized. This new sensor can be used to study the complex deformation behaviors of HMA;
b. In the standard DM tests and modified DM tests, LA-S had the best high temperature performance among the four asphalt mixtures, showing the largest DM and the smallest PA at 50 °C, of which the variation amplitude of mechanical parameters was the smallest. In addition, the DPR and phase lag of LA-S still showed the lowest value and change range;
c. The stiffness and PRS indices in the RLPD tests presented that the performance of the four HMAs can be ordered as LA-S > BA-S > BA-P > LA-P, which was consistent with the results evaluated by DM, PA, and CRP. From the perspective of DPR, the lateral deformation feature can reflect the high temperature stability and deformation resistance of the material. The smaller the value, the better the performance of the material;
d. In the RLPD tests, the total strain of the mixes was decomposed into three parts, being the elastic strain, viscoelastic strain, and viscoplastic strain. The RLSLS indices of different strain types presented the trend of $v_{e}<v_{ev}<v_{vp}$. The $v_{tatal}$ and $v_{e}$ values were generally less than 0.5, while the values of $v_{ve}$ and $v_{vp}$ could exceed 0.5;
e. The RLSLS index of $v_{tatal}$ can be used to comprehensively evaluate the visco-elasto-plastic behavior of the HMA, and the $R^{2}$ value of the linear fitting with the permanent deformation $\varepsilon_{vp}$ was approximately 0.75;
f. For different asphalt mixtures, the high temperature performance, mechanical properties; and deformation behavior were the different expressions of the constitutive relationship of the material. It made the various material parameters, DM, PA, CPR, stiffness, PRS; and RLSLS obtained based on different tests, finally show consistent results.

In order to establish an effective evaluation method and prediction model for flow-rutting, the relationship between the RLSLS index and the permanent deformation in a specific pavement structure under complex temperature and stress field should be further quantitated.

## Figures and Tables

**Table 1:** Physical properties of BA and LA volcanic ashes.

Filler	BA	LA
Average particle diameter (nm)	453	1901
Diameter (nm)/Proportion of the dominant particles	294/98.2%	1963/98.8%
Bulk specific surface area (m^2^/cm^3^)	4.94	114.88
Apparent density (g/cm^3^)	2.40	2.55

**Table 2:** The physical and mechanical properties of base asphalt.

Index	Results (units)	Specification
Penetration (25 °C) ^1^	99.0 (0.1 mm)	ASTM D5
Softening point	45.0 (°C)	ASTM D36
Ductility (10 °C, 5 cm/min) ^2^	78.0 (cm)	ASTM D113

Note: ^1^ The same batch of base asphalt was used for the SBS-modified asphalt binder. Penetration tests were performed at 25 °C. ^2^ The ductility of asphalt was taken at 10 °C instead of 25 °C as stipulated in ASTM D113 due to the low annual average temperature in Jilin area (China), between 40° and 44° north latitude.

**Table 3:** The composition of mixtures with different fine fillers and asphalt binders.

Asphalt Mixture	Volcanic Ash Fine Filler	Asphalt Binder
LA-S	LA	5 wt% SBS modified asphalt
LA-P		Base asphalt
BA-S	BA	5 wt% SBS modified asphalt
BA-P		Base asphalt

**Table 4:** The grading of aggregates.

Sieve Size (mm)	Percent Passing by Weight
19	100
16	92.6
13.2	82.7
9.5	68.2
4.75	49.1
2.36	32.4
1.18	23.6
0.6	17
0.3	12.7
0.15	9.0
0.075	6.6

**Table 5:** The details of the analyzed indices of different testing methods.

Testing Method	Index	Temperature	Axial Strain Level	Sensor Direction
Standard DM test	DM, PA (mechanics parameter)	35 °C, 50 °C	<200 με (viscoelastic behavior)	Axial
Modified DM test	CPR(DPR and phase lag) (mechanics parameter)	35 °C, 50 °C	<200 με (viscoelastic behavior)	Axia Lateral
RLPD test	Stiffness, RPS (performance index) RLSLS (performance index)	35 °C, 50 °C	400~600 με (elasto-visco-plastic behavior)	Axial Lateral

**Table 6:** The value range of DM and PA for the four mixtures at 35 °C and 50 °C.

Material	Index	Value Range (35 °C)	Value Range (50 °C)
LA-S	Dynamic modulus (MPa)	584~4482	317~1540
BA-S		447~4254	207~1262
LA-P		381~4869	155~1298
BA-P		319~3735	142~913
LA-S	Phase angle (degree)	28.2~34.7	21.0~34.3
BA-S		29.5~36.1	21.2~35.8
LA-P		31.2~39.2	21.9~39.1
BA-P		29.0~37.3	18.8~37.2

**Table 7:** The value range of DPR and phase lag of the four mixtures at 35 °C and 50 °C.

Material	Index	Value Range (35 °C)	Value Range (50 °C)
LA-S	DPR	0.18~0.24	0.22~0.28
BA-S		0.22~0.26	0.43~0.42
LA-P		0.32~0.35	0.46~0.53
BA-P		0.33~0.34	0.42~0.45
LA-S	Phase lag (degree)	4.17~9.98	2.30~4.71
BA-S		4.62~9.55	5.56~6.74
LA-P		10.85~14.70	5.45~6.98
BA-P		7.21~11.16	6.20~7.87

**Table 8:** The value range of RPS for the four mixtures at 35 °C and 50 °C.

Material	Sensor Direction	Value Range (35 °C)	Value Range (50 °C)
LA-S	Vertical RPS (με/s)	0.24~1.30	0.22~1.59
BA-S		0.25~2.07	0.47~6.02
LA-P		0.58~3.24	0.89~9.72
BA-P		0.59~3.24	0.75~4.85
LA-S	Horizontal RPS (με/s)	0.11~0.67	0.11~1.33
BA-S		0.14~1.31	0.24~4.17
LA-P		0.49~3.25	0.97~9.70
BA-P		0.53~3.00	0.89~7.54

**Table 9:** The order of different indices according to high-temperature performance.

Index	Testing Method	High Temperature Performance
DM, PA	Standard DM test	LA-S > BA-S > BA-P > LA-P
CPR	Modified DM test	LA-S > BA-S > BA-P > LA-P
Stiffness	RLPD test	LA-S > BA-S ≈ LA-P > BA-P
RPS	RLPD test	LA-S ≈ BA-S > LA-P ≈ BA-P
RLSLS	RLPD test	LA-S > BA-S > BA-P > LA-P

## Data Availability

Not Applicable.

## References

[1] Neifar M., Di Benedetto H. (2001). Thermo-viscoplastic law for bituminous mixes. Road Mater. Pavement Des..

[2] Di Benedetto H., Mondher N., Sauzéat C., Olard F. (2007). Three-dimensional thermo-viscoplastic behaviour of bituminous materials: The DBN model. Road Mater. Pavement Des..

[3] Zhang Q.-S., Chen Y.-L., Li X.-L. Rutting in asphalt pavement under heavy load and high temperature. Proceedings of the Asphalt Material Characterization, Accelerated Testing, and Highway Management: Selected Papers from the 2009 GeoHunan International Conference.

[4] İskender E. (2013). Rutting evaluation of stone mastic asphalt for basalt and basalt–limestone aggregate combinations. Compos. Part B.

[5] Pasquini E., Canestrari F., Cardone F., Santagata F. (2011). Performance evaluation of gap graded asphalt rubber mixtures. Constr. Build. Mater..

[6] Zhao S., Huang B., Shu X., Ye P. (2014). Laboratory investigation of biochar-modified asphalt mixture. Transp. Res. Rec..

[7] Zhao S., Huang B., Ye X.P., Shu X., Jia X. (2014). Utilizing bio-char as a bio-modifier for asphalt cement: A sustainable application of bio-fuel by-product. Fuel.

[8] Movilla-Quesada D., Muñoz O., Raposeiras A.C., Castro-Fresno D. (2018). Thermal suspectability analysis of the reuse of fly ash from cellulose industry as contribution filler in bituminous mixtures. Constr. Build. Mater..

[9] Lagos-Varas M., Movilla-Quesada D., Raposeiras A.C., Arenas J.P., Calzada-Perez M.A., Vega-Zamanillo A., Lastra-Gonzalez P. (2020). Influence of limestone filler on the rheological properties of bituminous mastics through susceptibility master curves. Constr. Build. Mater..

[10] Liu X., Liu W., Wang S., Wang Z., Shao L. (2018). Performance evaluation of asphalt mixture with nanosized volcanic ash filler. J. Transp. Eng. Part B Pavements.

[11] Liu X., Zhang M., Shao L., Chen Z. (2018). Effect of volcanic ash filler on thermal viscoelastic property of SBS modified asphalt mastic. Constr. Build. Mater..

[12] Van Thanh D., Feng C.P. (2013). Study on Marshall and Rutting test of SMA at abnormally high temperature. Constr. Build. Mater..

[13] Chaturabong P., Bahia H.U. (2017). Mechanisms of asphalt mixture rutting in the dry Hamburg Wheel Tracking test and the potential to be alternative test in measuring rutting resistance. Constr. Build. Mater..

[14] Wen H., Wu S., Mohammad L.N., Zhang W., Shen S., Faheem A. (2016). Long-term field rutting and moisture susceptibility performance of warm-mix asphalt pavement. Transp. Res. Rec..

[15] Zhang W., Shen S., Wu S., Mohammad L.N. (2017). Prediction model for field rut depth of asphalt pavement based on Hamburg wheel tracking test properties. J. Mater. Civ. Eng..

[16] Suh Y.-C., Cho N.-H., Mun S. (2011). Development of mechanistic–empirical design method for an asphalt pavement rutting model using APT. Constr. Build. Mater..

[17] Ji X., Zheng N., Niu S., Meng S., Xu Q. (2016). Development of a rutting prediction model for asphalt pavements with the use of an accelerated loading facility. Road Mater. Pavement Des..

[18] Tian Y., Lee J., Nantung T., Haddock J.E. (2017). Development of a mid-depth profile monitoring system for accelerated pavement testing. Constr. Build. Mater..

[19] Lagos-Varas M., Movilla-Quesada D., Arenas J.P., Raposeiras A.C., Castro-Fresno D., Calzada-Pérez M.A., Maturana J. (2019). Study of the mechanical behavior of asphalt mixtures using fractional rheology to model their viscoelasticity. Constr. Build. Mater..

[20] Lagos-Varas M., Raposeiras A.C., Movilla-Quesada D., Arenas J.P., Castro-Fresno D., Muñoz-Cáceres O., Andres-Valeri V.C. (2020). Study of the permanent deformation of binders and asphalt mixtures using rheological models of fractional viscoelasticity. Constr. Build. Mater..

[21] Ali Y., Irfan M., Ahmed S., Ahmed S. (2017). Empirical correlation of permanent deformation tests for evaluating the rutting response of conventional asphaltic concrete mixtures. J. Mater. Civ. Eng..

[22] Witczak M.W. (2005). Simple Performance Tests: Summary of Recommended Methods and Database.

[23] Witzcak M.W. (2002). Simple Performance Test for Superpave Mix Design.

[24] Gandomi A.H., Alavi A.H., Mirzahosseini M.R., Nejad F.M. (2011). Nonlinear Genetic-Based Models for Prediction of Flow Number of Asphalt Mixtures. J. Mater. Civ. Eng..

[25] Li Q., Yang H., Ni F., Ma X., Luo L. (2015). Cause analysis on permanent deformation for asphalt pavements using field cores. Constr. Build. Mater..

[26] (2006). ASTM, D6927-06.

[27] Zhou Z., Liu W., Huang Y., Wang H., He J., Huang M., Ou J. (2012). Optical fiber Bragg grating sensor assembly for 3D strain monitoring and its case study in highway pavement. Mech. Syst. Signal Processing.

[28] Liu W., Wang B., Zhou Z., Cao D., Zhao Y. (2017). Design and Testing of a Large-Scale Shape-Monitoring Sensor Based on Fiber-Bragg-Grating Sensing Technique for Pavement Structure. J. Transp. Eng. Part A Syst..

[29] (2007). AASHTO, TP62-07.

[30] Zhao Y., Richard Kim Y. (2003). Time–temperature superposition for asphalt mixtures with growing damage and permanent deformation in compression. Transp. Res. Rec..

[31] Airey G.D., Rahimzadeh B., Collop A.C. (2003). Viscoelastic linearity limits for bituminous materials. Mater. Struct..

[32] Di Benedetto H., Olard F., Sauzéat C., Delaporte B. (2004). Linear viscoelastic behaviour of bituminous materials: From binders to mixes. Road Mater. Pavement Des..

[33] (2015). AASHTO, TP79-15.

